# Bilateral posterior fracture-dislocation of the shoulder: Report of two cases

**DOI:** 10.4103/0973-6042.57935

**Published:** 2009

**Authors:** Rui Claro, Ricardo Sousa, Marta Massada, Joaquim Ramos, José M. Lourenço

**Affiliations:** Orthopaedic Department of Centro Hospitalar do Porto - Hospital de Santo António, Portugal

**Keywords:** Convulsive seizure, electrocution, posterior fracture-dislocation shoulder

## Abstract

Bilateral posterior fracture-dislocation of the shoulder is a very rare injury. Almost 50% of bilateral posterior dislocations are due to a convulsive seizure, rising to 90% if the dislocations are associated with fractures. Electric shock accounts for less than 5% of bilateral posterior dislocations of the shoulder. A systematization of the clinical and radiological approach, followed by an early diagnosis and proper surgical treatment is essential. Authors report 2 cases of bilateral posterior fracture-dislocation of the shoulder, one caused by a convulsive seizure and the other by an electric shock. A review of literature and a treatment protocol are also presented.

## INTRODUCTION

Although the shoulder is the most frequently dislocated joint, posterior dislocation is uncommon. Of all shoulder dislocations, 4% are posterior and 1% are associated with fractures.[1]

Bilateral posterior dislocation of the shoulder is a rare situation, representing less than 5% of all posterior dislocations.[2] Posterior fracture-dislocation of the shoulder is even less common, representing 0.9% of the 1500 dislocations and fracture-dislocations reviewed by Neer.[3,4]

Different aetiologies are attributed to this particular condition. The "triple E syndrome" (epilepsy or any convulsive seizure, extreme trauma and electric shock) stands for the 3 most frequent causes of bilateral posterior dislocation of the shoulder.[1] Almost 50% of bilateral posterior dislocations are due to a convulsive seizure, rising to 90% if the dislocations are associated with fractures.[5] Electric shock accounts for less than 5% of bilateral posterior dislocations of the shoulder.[1]

We present 2 cases of bilateral posterior fracture-dislocation of the shoulder, one caused by a convulsive seizure and the other by an electric shock, presented to our clinic over a period of 3 years.

## CASE REPORTS

### Case 1

A 36-year-old man, with a dominant right hand, a farm worker, with a history of alcohol abuse was hospitalized after an electric shock. He had bilateral burns in his hands and painful stiff shoulders. Functions (sensory and motor) of axillary nerve and all peripheral nerves were tested and were normal. Findings from vascular examination were also normal.

**Figure 1:**
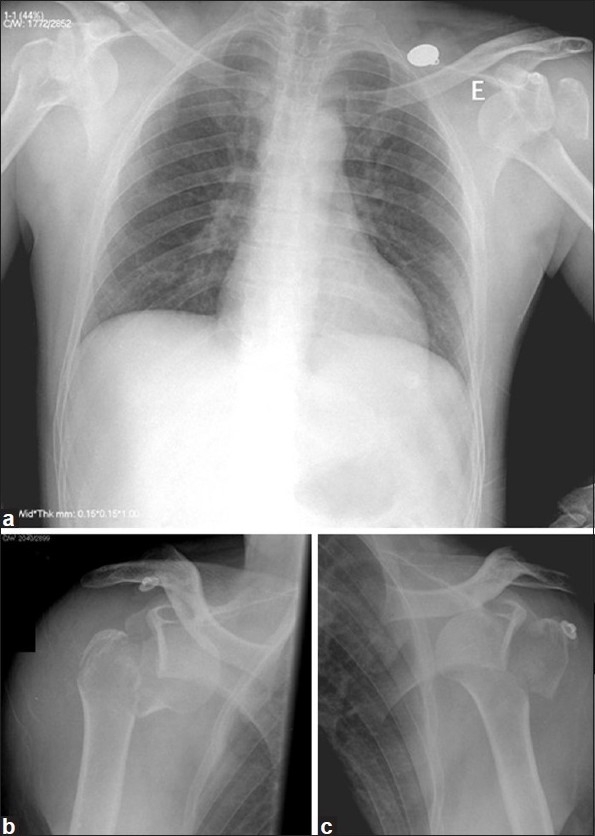
Radiographs of both shoulders (a) showing a posterior four-part fracture-dislocation of the right shoulder (b) and a posterior two-part fracture-dislocation of the left shoulder (c)

Plain anteroposterior and lateral radiographs of both shoulders showed a posterior four-part fracture-dislocation of the right shoulder and a posterior two-part fracture-dislocation of the left shoulder [Figure 1]. Although important and included in our protocol, the CT scan was not required by the colleague at the emergency room and the patient was taken to the operating room.

**Figure 2:**
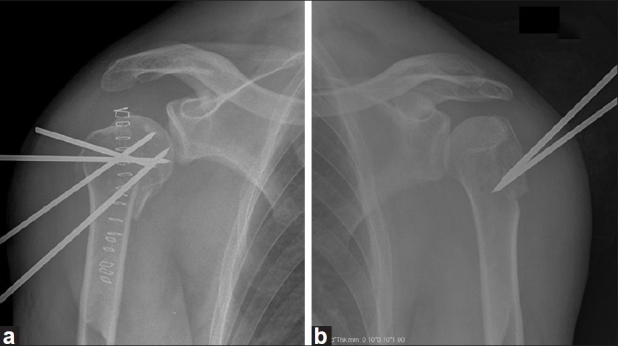
Radiographs (AP) of both [right- (a); left- (b)] shoulders 4 weeks after closed reduction under general anaesthesia and pin fixation with K-wires

The patient was submitted to gentle closed reduction under general anaesthesia of both shoulders. There was an acceptable anatomical relationship between the fragments and pin fixation with K-wires was made to maintain the fracture alignment [Figure 2].

Postoperatively both shoulders were immobilised for 4 weeks with a sling. Pins were removed after that, and active rehabilitation was started.

**Figure 3:**
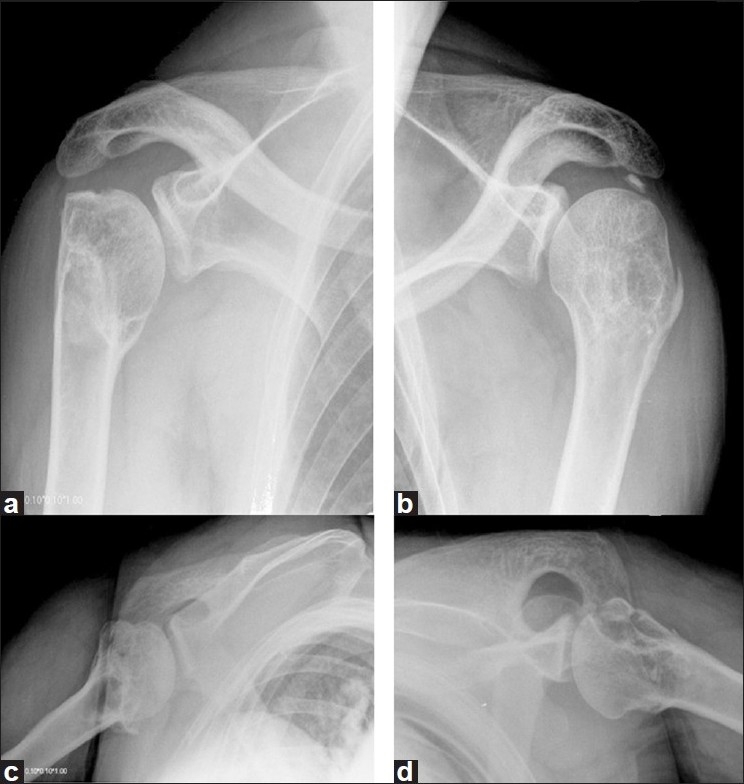
Radiographs of both shoulders [right- (a,c); left- (b,d)] 27 months after trauma, showing consolidation of the fractures without signs of avascular necrosis

Twenty-seven months after the trauma, the patient has painless shoulders, has returned to his previous level of work and is very satisfied with the results regarding functions of the shoulder: flexion on the left side, 130°; flexion on the right side, 120°; abduction on the left side, 120°; abduction on the right side, 120°; external rotation on the left side, 40°; external rotation on the right side, 40°; internal rotation on the left side, L3; internal rotation on the right side, T12. The final Constant score[6] was 71 on the left side and 69 on the right side. Results of apprehension tests of both shoulders were negative. Radiographs showed consolidation of the fractures without signs of avascular necrosis [Figure 3].

### Case 2

A 57-year-old housemaid, with a dominant right hand, was hospitalized after an acute major convulsive seizure. The patient had very painful shoulders, and the functions (sensory and motor) of the axillary nerve and peripheral nerves were normal. Findings from vascular examination were also normal.

**Figure 4:**
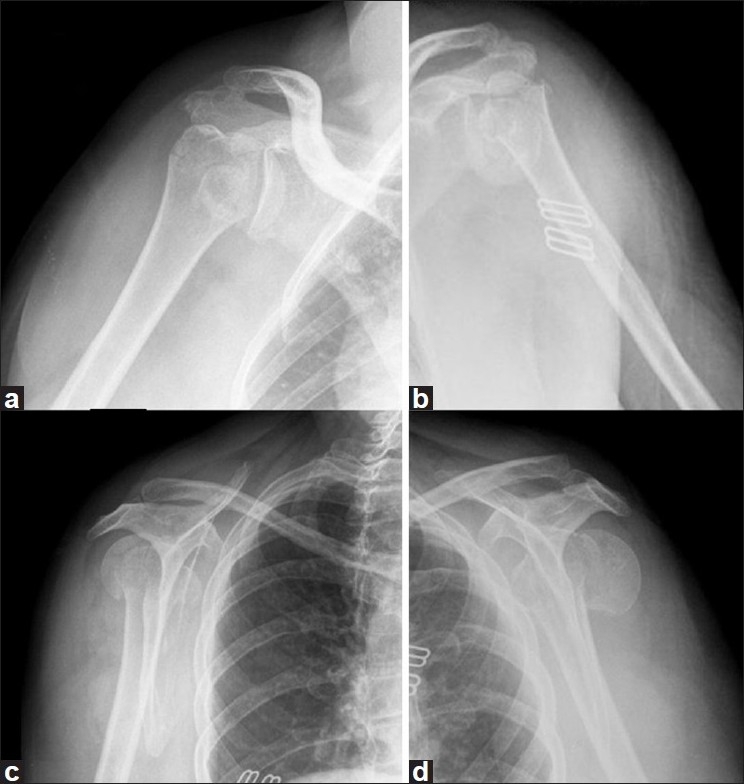
Radiographs of both shoulders [right- (a,c); left- (b,d)] showing a bilateral posterior four-part fracture-dislocation of the shoulderr

**Figure 5:**
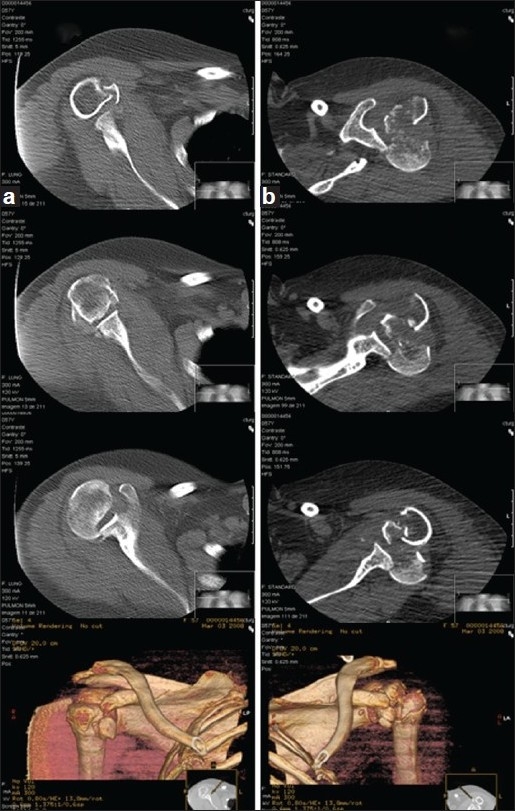
CT scan of both shoulders [right- (a); left- (b)] confirming a bilateral posterior four-part fracture-dislocation of the shoulder

Plain anteroposterior and lateral radiographs of both shoulders showed bilateral comminute proximal humeral fractures [Figure 4]. She also underwent a CT scan, which confirmed a bilateral posterior four-part fracture-dislocation of the shoulder [Figure 5].

Attempts to carry out closed reduction under general anaesthesia of both shoulders were unsuccessful. The patient was submitted to open reduction of both sides, using a deltopectoral approach. At the right side an acceptable reduction of the fragments with a locking plate was achieved. At the left side the fragments were very small, displaced and without structural support to maintain the reduction, so at the left shoulder a hemiprosthesis was used.

Postoperatively both shoulders were immobilized for 4 weeks with a sling; and after that, active rehabilitation was started. The patient had complaints of painful right shoulder during the first 3 months, with progressively better functional results.

**Figure 6:**
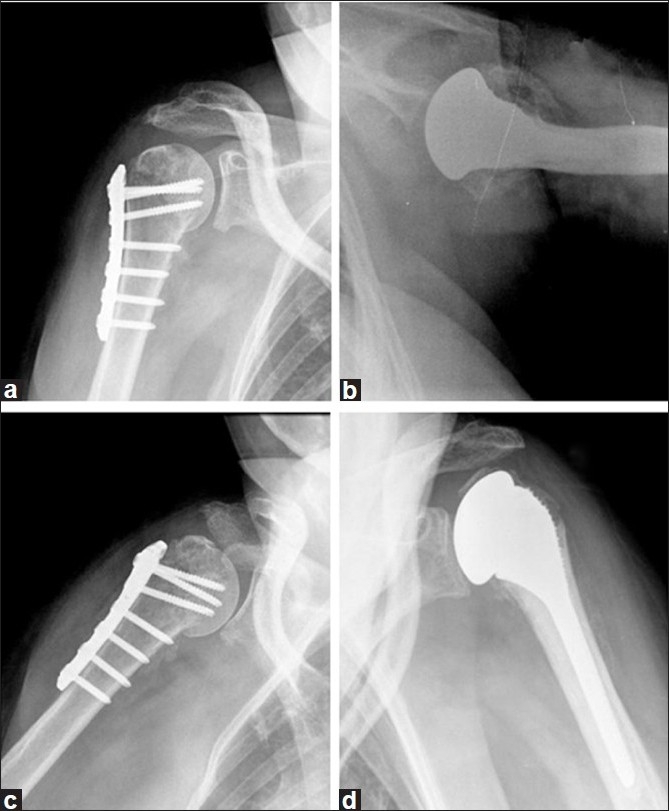
Radiographs of both shoulders 15 months after injury, showing consolidation of the right shoulder fracture (a,c) without signs of avascular necrosis, and good positioned hemiarthroplasty on the left shoulder (b,d) without evidence of loosening

Fifteen months after the trauma, the patient has painless shoulders, has returned to her previous level of work and is satisfied with the results regarding functions of the shoulder: flexion on the left side, 130°; flexion on the right side, 100°; abduction on the left side, 110°; abduction on the right side, 100°; external rotation on the left side, 30°; external rotation on the right side, 30°; internal rotation on the left side, L3; internal rotation on the right side, L3. The final Constant score[6] was 71 on the left side and 63 on the right side. Results of apprehension tests of both shoulders were negative. Radiographs showed consolidation of the fractures without signs of avascular necrosis on the right side, and a well-positioned hemiarthroplasty on the left side without evidence of loosening [Figure 6].

## DISCUSSION

Bilateral posterior fracture-dislocation of the shoulder, first described in 1902 by Mynter,[1] is extremely rare. The most common cause for this situation is a convulsive seizure.[7]

The proposed mechanism of shoulder injury during a convulsive seizure has been well described.[8] The typical position of the shoulder during a convulsion is adduction, internal rotation and flexion. With the spasm the humeral head is forced superiorly and posteriorly over the glenoid cavity. If the convulsion stops, the humeral head stays locked behind the glenoid , often with a reversed Hill-Sachs lesion. Further convulsive force, with the humeral head impinged against the glenoid rim, results eventually in a complex proximal humeral fracture.

Diagnosis of this type of injury is often delayed,[9,10] up to 50% not being correctly identified at the first presentation.[10] especially in a patient with a first episode of convulsive seizure who wakes up with painful stiff shoulders without memory of any trauma.[5]

Accurate history-taking and physical examination with at least one anteroposterior and one axillary radiograph views[2] are essential when assessing any shoulder complaint. At the time of physical examination shoulder contour may be normal with a prominent coracoid process. Painful abnormal movement at the fracture site may be mistaken for normal glenohumeral movement. Attention should be directed towards associated nerve injuries. A CT scan also provides also a complete description of the lesion and can be important for planning surgery.[10]

Although it is included in our standard protocol, the CT scan was not done in the first case described above. The surgical team only used plain anteroposterior and lateral radiographs of both shoulders, which conditioned the preoperative planning.

Treatment should be adapted to the type of lesion, the interval of time between trauma and treatment, and also to the age, occupation and desired levels of activity of the patient.[5] Good results can be expected only if the anatomy is respected and if the procedure provides a stable joint.

When the fracture is minimally displaced and the viability of the humeral head is not in doubt,[11‐13] closed reduction, and if necessary pin fixation, should be done.[14,15] It has been stated that 3 weeks after trauma, closed reduction is almost impossible and surgical treatment is required.[16]

For displaced acute fractures in young patients, if an attempt of gentle closed reduction is not successful, open reduction and internal fixation is the option. If open reduction cannot be achieved or in cases in which more than 50% of the articular surface of the head is damaged, hemiarthroplasty is a good solution.

In older patients (>65 years old) with three or four-part acute fractures, there is a high risk of avascular necrosis, and the option is hemiarthroplasty.[5,7,17] In patients over 70 years of age with locked dislocation or fracture-dislocation associated with rotator cuff tear and severe distortion of the bony anatomy, a reverse prosthesis is likely to provide good results.[18]

If there is involvement of humeral head and also glenoid damage, a total shoulder arthroplasty may be considered.[19]

**Figure 7:**
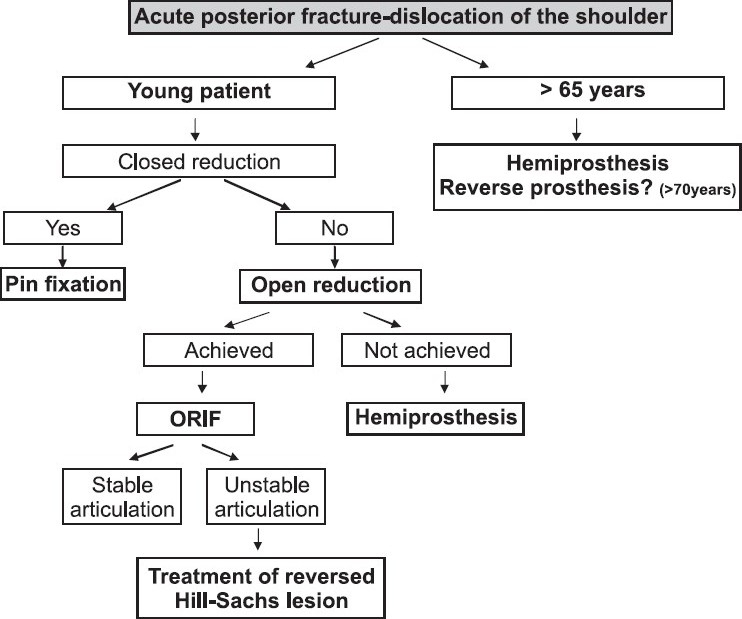
Acute posterior fracture-dislocation of the shoulder

Taking into account the above, we base our approach on the treatment algorithm [Figure 7] we found to be the most suitable for this particular situation. In the first case described above, a successful gentle closed reduction was achieved on the left side, so an attempt of closed reduction was also made on the right side (posterior four-part fracture-dislocation), where also there was an acceptable reduction, although not expected due to the complexity of the fracture. In the second case, both shoulders presented comminute-proximal humeral fractures, and they could be managed only with open reduction and internal fixation on the right side and with a hemiprosthesis on the left side.

When the diagnosis is delayed for several weeks or months and when when there is no evidence of avascular necrosis, other procedures can be preformed: humeral osteotomy to correct mal-union and a McLaughlin's procedure[20‐22] or auto/allograft reconstruction of the humeral head defect (residual reversed Hill-Sachs lesion)[23,24] to maintain reduction of the posterior dislocation. Some authors agree, depending on preoperative and intra-operative findings, about using shoulder replacement when duration of the dislocation is longer than 6 months.[16,19,25‐27] Although bilateral posterior fracture-dislocation of the shoulder is a very rare lesion, functional limitation of both shoulders can be devastating. A constant systematization of the clinical approach and radiological study of the traumatized shoulders is essential. Early diagnosis and proper surgical treatment, performed by an experienced shoulder surgeon can maximize final functional results.
